# Genetic Predictors of Early-Onset Spinal Intervertebral Disc Degeneration: Part One of Two

**DOI:** 10.7759/cureus.15182

**Published:** 2021-05-22

**Authors:** Brian Fiani, Claudia Covarrubias, Ryan Jarrah

**Affiliations:** 1 Neurosurgery, Desert Regional Medical Center, Palm Springs, USA; 2 Medicine, Universidad Anahuac Queretaro, Santiago de Querétaro, MEX; 3 Miscellaneous, University of Michigan - Flint, Flint, USA

**Keywords:** aggrecan, collagen, matrix metalloproteinase, vitamin d3 receptor, interleukin, spinal degenerative disease

## Abstract

Intervertebral disc (IVD) degeneration is a progressive and painful pathology that can root from mechanical, biochemical, and environmental stressors. However, recent advancements in biogenetics have now found a predominating genetic influence. Nevertheless, despite these advancements, the pathophysiology of IVD degeneration remains poorly understood. In the first of our two-part series, we will characterize some of the most recent and best-studied genes in the context of intervertebral disc degeneration. We will attempt to formulate the first contemporary gene guide that characterizes the genetic profile of IVD degeneration. The genes of interest include aggrecan (ACAN), matrix metalloproteinase 2 (MMP2), vitamin D receptor (VDR), interleukin 1 alpha (IL1A), and those encoded for collagens such as collagen type XI alpha 1 chain (COL11A1), collagen type I alpha 1 chain (COL1A1), collagen type IX alpha 2 chain (COL9A2), and collagen type IX alpha 3 chain (COL9A3). Genetic analysis studies reveal that these genes play vital roles in maintaining the structural integrity of the intervertebral disc, activating enzymes involved in the extracellular matrix, and promoting connective tissue formation. Nevertheless, characterizing these genes alone is not enough to understand the pathophysiology of IVD degeneration. Therefore, further studies are warranted to understand molecular signalling pathways of IVD degeneration better and ultimately create more sophisticated genetic and cell-based therapies to improve patient outcomes.

## Introduction and background

The intervertebral disc (IVD) is among the most crucial biological structures in allowing for the functions of everyday life. IVDs are avascular and fibrocartilaginous structures that maintain the structural and functional integrity of the vertebrae. In addition, IVD’s transmit compressible loads, act as a shock absorber, and allow for spinal flexibility [1]. The IVD is composed of two structural components: the annulus fibrosis (AF) and the nucleus pulposus (NP) [2]. The AF is the outer fibrous segment that serves a role in constraining the mobility of the IVD [3]. The NP is the inner gelatinous core that maintains the flexibility of the spine [1]. Both the AF and NP serve in maintaining homeostasis of the spine; however, these components can degrade over time leading to what is clinically known as intervertebral disc degeneration [1]. IVD degeneration is a painful and progressive disease characterized by the breakdown of the IVD, causing hallmark signs of neck and back pain [4]. With neck and back pain being among the leading global causes of disability, understanding the pathophysiology of IVD degeneration paramount to restoring and preserving the functional lives of diagnosed patients.

Unfortunately, despite extensive research, there is a significant knowledge gap surrounding the pathophysiology of IVD degeneration [5]. However, recent advancements in biogenetics have found a profound genetic component to its clinical presentation. Over the past two decades, an increasing number of identified unregulated genes promote inflammatory and apoptotic enzymes and growth factors that facilitate degeneration of the IVD [6]. Moreover, mutations and polymorphisms to structural genes have also catalyzed the breakdown of the AF and NP, leading to a more complicated treatment course [7]. In the first to our two-part review on IVD genes, we will characterize some of the recent and best-studied genes secondary to IVD degeneration. In doing so, we will compose the first compressive gene guide for IVD degeneration to establish genetic markers that could indicate the occurrence of IVD degeneration.

## Review

Genetic Analysis

Various association and linkage analyses studies have identified multiple genes attributed to the initiation and progression of IVD degeneration [8,9]. Amongst the pool of genes mentioned in the literature, we decided to focus on a total of 13 genes divided into a two-part series. For the first segment, we will elaborate on eight genes that possess a strong level of interaction with the structural components of the IVD and the latest research associating them to the topic (Table 1).

**Table 1:** Characteristics of genetic factors associated with disc degeneration. Abbreviations: USA- United States of America, VNTR- variable number of tandem repeats, DDD- degenerative disc disease, OR- odds ratio, CI- confidence interval, p- p-value, SNP- single nucleotide polymorphism, LDH- lumbar disc herniation, IL-1⍺- interleukin 1 alpha, IDD- intervertebral disc degeneration, MMP- matrix metalloproteinase, ECM- extracellular matrix, MMP2- matrix metallopeptidase 2, a- systematic review and meta-analysis

Gene name		Genomic region	Encoded-protein family	Selected studies	Number of studies included^a^	Countries or ethnicities included	Findings
ACAN	aggrecan	15q26.1	aggrecan/versican proteoglycan family	Cong et al. 2018 [10] ^a ^	5	China, Turkey, USA, Japan, South Korea, Finland	VNTR polymorphism on allele 21 was over-represented and was found to increase the risk of DDD.
COL11A1	collagen type XI alpha 1 chain	1p21.1	type XI collagen	Liu et al. 2017 [11]	N/A	China	SNP rs1676486 may be functionally associated with LDH.
COL1A1	collagen type I alpha 1 chain	17q21.33	type I collagen	Hanaei et al. 2020 [12]	N/A	Iran	SNP rs909102 was not significantly associated with DDD.
				Pluijm et al. 2004 [13]	N/A	Netherlands	COLIA1 Sp1 polymorphism may be beneficial for the prediction of DDD in older patients.
COL9A3	collagen type IX alpha 3 chain	20q13.33	type IX collagen	Huang et al. 2018 [14] ^a^	11	Iran, Finland, Greece, USA, India, China, Turkey	COL9A3 trp3 polymorphism did not seem to be connected to the risk of IDD in any gender, continent or ethnicity of people.
				Wu et al. 2018 [15] ^a^	10	Finland, Japan, China, South Korea, India, Denmark	COL9A3 gene (rs61734651) and COL9A2 gene (rs12077871, rs12722877, rs7533552) polymorphisms were not associated with susceptibility to LDD.
COL9A2	collagen type IX alpha 2 chain	1p34.2		Hanaei et al. 2020 [12]	N/A	Iran	COL9A2 rs137853213 was not significantly associated with DDD.
IL1A	interleukin 1 alpha	2q14.1	IL 1 cytokine family	Ahn et al. 2002 [16]	N/A	South Korea	Suggests that IL-1⍺ exists in herniated discs but does not seem to be an abundant proinflammatory cytokine.
				Chen. et al. 2018 [17]	N/A	China	IL-1α -889C/T polymorphism was associated with an increased risk of IDD.
MMP2	matrix metalloproteinase 2	16q12.2	zinc-dependent proteinase family	Zhang et al. 2013 [18]	N/A	China	The -735 C/T polymorphism of MMP2 may be associated with the risk and severity of LDD.
				Dong et al. 2007 [19]	N/A	China	The frequency of the MMP-2 -1306CC genotype was significantly higher in patients with LDD than in the healthy population (26) A threefold increased risk for LDD was also estimated with the CC genotype.
VDR	vitamin D receptor	12q13.11	nuclear hormone receptor superfamily of ligand-inducible transcription factors	Pekala et al. 2018 [20] ^a^	7	Caucasian, Hispanic, Asian	There is no evidence of an association between the FokI (rs2228570) polymorphism and IDD in the general population. Ethnic-specific analyses show that Caucasians with FokI have decreased risk of IDD, while Hispanics with FokI have significantly higher risk of IDD.
				Jiang et al. 2016 [21] ^a^	23	Caucasian, Asian	TaqI, FokI, and ApaI polymorphisms of the VDR gene were not significantly associated with the predisposition of LDD.

Aggrecan

The aggrecan gene (ACAN) is a member of the aggrecan and versican proteoglycan family that encodes a major proteoglycan component of the hyaline cartilage and the nucleus pulposus (NP) in the form of chondroitin sulfate and keratan sulfate chains, respectively [22,23]. It is directly responsible for maintaining disc hydration, propagated by the covalent bonding of anionic glycosaminoglycans, and thus the load-bearing and shock-absorbing functions of the IVD [9,22]. Hence, the loss of ACAN gene function, resulting in disc dehydration, has been identified as an early indicator of disc degeneration and a potentiator of NP herniation [22,24]. The ACAN gene consists of two domains, CS1 and CS2, where a VNTR polymorphism in the CS1 gene domain, located in exon 12, has proven to result in variant ACAN structures propagating the loss of function and ultimate IVDD [5]. A systematic review on the relationship between the distributions of ACAN gene VNTR polymorphism and IVD degeneration was conducted by Cong et al. in 2018 with seven studies, including five only pertaining to DDD included Caucasian, Asian, and Turkish populations [10]. Cong et al. concluded that the over-representation of ACAN gene VNTR allele 21 increased the risk of DDD [10]. A year later, Yaltirik et al. published a study concluding that the ACAN gene c.6423T>C variant was not correlated with the development and severity of LDDD on the Turkish population [22]. These inconsistent findings can be attributed to unknown interactions between other associated genes and the differences found within the distribution of alleles between ethnic groups [10].

Collagens

The extracellular matrix (ECM) of the IVD is structurally composed of proteoglycans, glycoproteins, polysaccharides and fibres such as collagens that regulate homeostasis [25]. Collagen is the most abundant protein in the ECM that function as structural support. There are 28 different collagens identified within the ECM, abounding in collagen types I, II, III, IV, V, IX, X, and XI [25,26]. Each collagen molecule contains a total of three polypeptide chains that are distributed in a complex, triple helix arrangement pre-determined by genetic predisposition [25]. Given that disc degeneration mainly affects the homeostasis of the ECM, several single nucleotide polymorphisms (SNPs) of these proteins have been studied extensively to identify a concrete association to the etiology and pathology of IVDD. Collagen I alpha 1 (COL1A1), collagen IX alpha-1 chain (COL9A1) and alpha-3 chain (COL9A3), and collagen type XI alpha-1 chain (COL11A1) have all been implicated in the pathophysiology of IVDD [15].

Fibrillar Collagen

Collagen I

Collagen I creates a network of fibres keeping together the NP and is also considered the main protein in bone, skin, and the outer layer of the FA [25]. The genes encoding collagen type I, COL1A1 and COL1A2, are present in both NP and FA. Although the mechanism by which genetic alterations of collagen I influence IDD development is not yet fully understood, polymorphisms of the COL1A1 gene have been reported to increase IDD risk in different population studies [27,28]. COL1A1 Sp1 genotype increased the risk of disc degeneration in elderly Dutch patients [13]. In contrast, a more recent study in the Iranian population concluded that the allele and genotype distributions of COL1A1 rs909102 and COL9A2 rs137853213 SNPs were not significantly associated with IVDD [26].

Collagen XI

Type XI collagen is another cartilage-specific ECM protein found in both the AF and NP of the IVD. Type XI collagen is composed of three chains that COL11A1 encodes, COL11A2, and COL11A3, respectively. Studies have reported narrowing of the IVD due to mutations in the COL11A1 gene [11]. The SNP polymorphism rs1676486 of this gene and decreased expression of COL11A1 have been associated with susceptibility to lumbar disc herniation (LDH) and greater severity of DDD [11,29].

Non-Fibrillar Collagen

Collagen IX

The Collagen IX gene is generally described as a heterotrimeric protein that acts as a bridge between collagenous and non-collagenous proteins in normal tissues. It is considered the major collagen component of hyaline cartilage and is made up of three alpha chains, each encoded by a distinct gene [30]. Although the amount of collagen IX is scarce in both the AF and NP, inconclusive data demonstrates a weak association between polymorphisms in both the COL9A2 and COL9A3, encode ⍺2 and ⍺3 chains on collagen IX, respectively, and the risk of LDD [15]. A meta-analysis on the association of COL9A3 trp3 polymorphism with IVDD published in 2018 concluded no significant association between the polymorphism and IVDD [14].

Interleukin-1

Pro-inflammatory cytokines are also involved in ECM degradation and fibrosis of the IVD [31]. Interleukin-1 (IL-1) is an inflammatory cytokine that contributes to disc degeneration by activating degradative enzymes, such as MMPs, and inhibiting of proteoglycan resynthesis [9]. (17)IL-1 is produced by monocytes and macrophages in the form of a proprotein, which is proteolytically processed and released in response to cell injury, thus inducing apoptosis [32]. Moreover, the IL1A gene has been previously identified to carry a threefold risk of disc bulges compared to controls [9,33]. It has been shown that an increased level of the pro-inflammatory IL1A antagonizes IL-1 receptor antagonist (IL1RN) in the degenerated disc as there is a dysregulation about tissue destruction [27]. A population study conducted in China demonstrated an association between IL1 alpha- 889C/T polymorphism and increased risk of IDD. The same study combined a meta-analysis, further indicating a significant association in the overall populations [17]. Other interleukins such as IL-4, IL-10 and IL-6, not discussed in this review, have also been associated with IVDD [8,12].

Matrix Metalloproteinase-2 (MMP2)

MMP2 is a metalloproteinase gene encoding an enzyme belonging to a multidomain zinc-dependent proteinase family [34,35]. Multiple studies in both humans and animal models have documented MMP gene expression associated with catabolic changes in the IVDs ECM degradation process [34,36]. Twenty-three different MMPs are expressed in human tissue and are typically subdivided according to bioinformatic criteria or functional characteristics [35]. MMP2 is primarily a gelatinase, specifically gelatinase A, but can also function as a type IV collagenase. Furthermore, MMP2 contains three fibronectin type II repeats in its catalytic site that allow binding denatured type IV and V collagen and elastin. MMP2 is essentially a degeneration mediator that plays a significant role in bone remodelling and has been shown to exhibit an upregulated expression pattern in degenerative NP tissue [29,35]. In 2007, Dong et al. determined that the MMP-2 -1306CC genotype frequency was significantly higher in young Chinese patients with lumbar IDD than a healthy population and estimated a threefold increased risk for LDD the CC genotype [19]. (26)Additionally, Zhang et al. published a study in 2013 where they concluded that the -735 C/T polymorphism of MMP2 might be associated with the risk and severity of LDD in the Chinese population [18]. Currently, there is a lack of systematic reviews or meta-analysis correlating MMP2 expression about IVDD.

Vitamin D3 receptor

Vitamin D3 receptor (VDR) is a protein belonging to the nuclear steroid hormone-receptor family encoded by the VDR gene [37]. VDR plays a vital role in regulating metabolic pathways, immune response, and osseous mineralization and remodelling [9,20,37]. Through a series of case-control studies across various ethnic groups, the TaqI (rs731236), FokI (rs2228570), and ApaI (rs7975232) polymorphisms of the VDR gene have been associated with LDD risk [21]. About the TaqI polymorphism, several of these studies have demonstrated that the presence of the t allele increases the risk of IVDD [9,24]. Other studies have stated that the Fokl (rs2228570) polymorphism of VDR has been inconclusively associated with IDD [20]. Pekala et al. conducted a systematic review where the overall findings were not statistically significant in the association between IDD and the Fokl polymorphism. Nevertheless, ethnic predisposition was highlighted in the Hispanic population in the presence of dominant and dominant/homozygous/heterozygous models of the Fokl rs 2228670 polymorphism [20].

There are other genes not specified in our present study as the current evidence has yielded contradictory results rather than evidence that supports their direct influence on IVDD. Large-scale cohorts and well-designed studies focused on various ethnic entities are needed to further analyze these genes' role in IVDD. Clarification is warranted regarding the level of interaction between catabolic and anabolic genetic factors and the identification of upregulatory or suppression mechanisms within those interactions. Lastly, it is imperative to mention that although earlier data indicating association with certain genetic factors and DDD, newer studies have been unable to replicate statistically significant results.

## Conclusions

These genes all serve as significant biomarkers of IVD degeneration and the intervertebral environment. Nevertheless, while the characterization of these genes may further understand IVD degeneration's molecular complexity, the genetic understanding of IVD degeneration remains to be poorly understood. This is because IVD degeneration is a polygenic disease, with multiple genes contributing to its pathogenesis. The complexity of the genetic code also indicates that gene interactions can have epistatic effects that are not completely understood in IVD degeneration. This complicates the pathophysiological understanding of IVD degeneration and the use of cell-based and genetic therapies for future mainstream practice. Therefore, future research is warranted to identify more genes involved with IVD degeneration and find the molecular pathways and targets affected. In doing so, a more compressive understanding of IVD degeneration's pathophysiology can be formed to foster more sophisticated therapies and improve future patient outcomes.

## References

[1] Yoon SY (1983). A legacy without heirs: Korean indigenous medicine and primary health care. Soc Sci Med.

[2] Iu J, Massicotte E, Li SQ, Hurtig MB, Toyserkani E, Santerre JP, Kandel RA (2017). In Vitro generated intervertebral discs: toward engineering tissue integration. Tissue Eng Part A.

[3] Dobozy O, Csaba G, Deák BM (1983). Interaction of thyrotropin (TSH) and gonadotropins in the function of genital organs. effect of TSH and gonadotropin pretreatment (hormonal imprinting) of newborn rats on hormonal overlap in adult animals. Acta Physiol Hung.

[4] Haynes RE, Azimi PH, Cramblett HG, Hilty MD, Burech DL, Koranyi KI (1975). Clinically distinguishable syndromes caused by viruses. Curr Probl Pediatr.

[5] Munir S, Rade M, Määttä JH, Freidin MB, Williams FMK (2018). Intervertebral disc biology: genetic basis of disc degeneration. Curr Mol Biol Rep.

[6] Fiani B, Jarrah R, Cathel A, Sarhadi K, Covarrubias C, Soula M (2021). The role of gene therapy as a valuable treatment modality for multiple spinal pathologies. Regen Med.

[7] Fiori A, Marigo M (1967). A method for the detection of d-tubocurarine, gallamine, decamethonium and succinylcholine in biological materials. Modification and development. J Chromatogr.

[8] Guan Y, Wang S, Wang J, Meng D, Wu H, Wei Q, Jiang H (2020). Gene polymorphisms and expression levels of interleukin-6 and interleukin-10 in lumbar disc disease: a meta-analysis and immunohistochemical study. J Orthop Surg Res.

[9] Kawaguchi Y (2018). Genetic background of degenerative disc disease in the lumbar spine. Spine Surg Relat Res.

[10] Cong L, Tu G, Liang D (2018). A systematic review of the relationship between the distributions of aggrecan gene VNTR polymorphism and degenerative disc disease/osteoarthritis. Bone Joint Res.

[11] Liu W, Sun G, Guo L (2017). A genetic variant in COL11A1 is functionally associated with lumbar disc herniation in Chinese population. J Genet.

[12] Hanaei S, Abdollahzade S, Sadr M (2020). The role of interleukin 4 and IL-4RA in intervertebral disc degeneration: investigation of single nucleotide polymorphisms in genes and a systematic review & meta-analysis of IL-4 expression level. Br J Neurosurg.

[13] Pluijm SM, van Essen HW, Bravenboer N, Uitterlinden AG, Smit JH, Pols HA, Lips P (2004). Collagen type I alpha1 Sp1 polymorphism, osteoporosis, and intervertebral disc degeneration in older men and women. Ann Rheum Dis.

[14] Huang D, Deng X, Ma K (2018). Association of COL9A3 trp3 polymorphism with intervertebral disk degeneration: a meta-analysis. BMC Musculoskelet Disord.

[15] Wu H, Wang S, Chen W, Zhan X, Xiao Z, Jiang H, Wei Q (2018). Collagen IX gene polymorphisms and lumbar disc degeneration: a systematic review and meta-analysis. J Orthop Surg Res.

[16] Ahn SH, Cho YW, Ahn MW, Jang SH, Sohn YK, Kim HS (2002). mRNA expression of cytokines and chemokines in herniated lumbar intervertebral discs. Spine (Phila Pa 1976).

[17] Chen Y, Ma H, Bi D, Qiu B (2018). Association of interleukin 1 gene polymorphism with intervertebral disc degeneration risk in the Chinese Han population. Biosci Rep.

[18] Zhang Y, Gu Z, Qiu G (2013). Association of the polymorphism of MMP2 with the risk and severity of lumbar disc degeneration in the Chinese Han population. Eur Rev Med Pharmacol Sci.

[19] Dong DM, Yao M, Liu B, Sun CY, Jiang YQ, Wang YS (2007). Association between the -1306C/T polymorphism of matrix metalloproteinase-2 gene and lumbar disc disease in Chinese young adults. Eur Spine J.

[20] Pekala PA, Henry BM, Taterra D, Piwowar M, Vikse J, Tubbs RS, Tomaszewski KA (2019). FokI as a genetic factor of intervertebral disc degeneration: a PRISMA-compliant systematic review of overlapping meta-analyses. J Clin Neurosci.

[21] Jiang H, Qin Z, Zong S, He M, Zhan X, Xiao Z, Wei Q (2017). Vitamin D receptor gene polymorphisms and lumbar disc degeneration: a systematic review and meta-analysis. Eur Spine J.

[22] Yaltirik CK, Timirci-Kahraman Ö, Gulec-Yilmaz S, Ozdogan S, Atalay B, Isbir T (2019). The evaluation of proteoglycan levels and the possible role of ACAN Gene (c.6423T&gt;C) variant in patients with lumbar disc degeneration disease. In Vivo.

[23] ACAN aggrecan (2021). ACAN aggrecan [Homo sapiens (human)]. https://www.ncbi.nlm.nih.gov/gene/176.

[24] Teles Filho RV, Abe GM, Daher MT (2020). Genetic influence in disc degeneration - systematic review of literature. Rev Bras Ortop (Sao Paulo).

[25] Laronha H, Caldeira J (2020). Structure and function of human matrix metalloproteinases. Cells.

[26] Hanaei S, Abdollahzade S, Sadr M, Fattahi E, Mirbolouk MH, Khoshnevisan A, Rezaei N (2021). Lack of association between COL1A1 and COL9A2 single nucleotide polymorphisms and intervertebral disc degeneration. Br J Neurosurg.

[27] Feng Y, Egan B, Wang J (2016). Genetic factors in intervertebral disc degeneration. Genes Dis.

[28] (2021). COL1A1 collagen type I alpha 1 chain [Homo sapiens (human)]. https://www.ncbi.nlm.nih.gov/gene/1277.

[29] (2021). COL11A1 collagen type XI alpha 1 chain [Homo sapiens (human)]. https://www.ncbi.nlm.nih.gov/gene/1301.

[30] (2021). COL9A2 collagen type IX alpha 2 chain [Homo sapiens (human)]. https://www.ncbi.nlm.nih.gov/gene/1298.

[31] Omair A, Holden M, Lie BA, Reikeras O, Brox JI (2013). Treatment outcome of chronic low back pain and radiographic lumbar disc degeneration are associated with inflammatory and matrix degrading gene variants: a prospective genetic association study. BMC Musculoskelet Disord.

[32] IL1A interleukin 1 alpha (2021). IL1A interleukin 1 alpha [Homo sapiens (human)]. https://www.ncbi.nlm.nih.gov/gene/3552.

[33] Djuric N, Lafeber GCM, Vleggeert-Lankamp CLA (2020). The contradictory effect of macrophage-related cytokine expression in lumbar disc herniations: a systematic review. Eur Spine J.

[34] Vo NV, Hartman RA, Yurube T, Jacobs LJ, Sowa GA, Kang JD (2013). Expression and regulation of metalloproteinases and their inhibitors in intervertebral disc aging and degeneration. Spine J.

[35] (2021). MMP2 matrix metallopeptidase 2 [Homo sapiens (human)]. https://www.ncbi.nlm.nih.gov/gene/4313.

[36] Tsai TT, Lai PL, Liao JC (2013). Increased periostin gene expression in degenerative intervertebral disc cells. Spine J.

[37] (2021). VDR vitamin D receptor [Homo sapiens (human)]. https://www.ncbi.nlm.nih.gov/gene/7421.

